# Cervical Disc Extrusion with Dorsal Migration in a Pet Rabbit

**DOI:** 10.3390/vetsci11070328

**Published:** 2024-07-21

**Authors:** Laura Porcarelli, Elena Dell’Era, Tommaso Collarile, Valeria De Palma, Noemi Morara, Kaspar Matiasek, Daniele Corlazzoli

**Affiliations:** 1 Centro Veterinario Gregorio VII, 00165 Rome, Italy; tommasocollarile@gregoriovii.com (T.C.); valeriadepalma@gregoriovii.com (V.D.P.); noemimorara@outlook.com (N.M.); danielecorlazzoli@gregoriovii.com (D.C.); 2 Section of Clinical & Comparative Neuropathology, Institute of Veterinary Pathology, Centre for Clinical Veterinary Medicine, LMU Munich, 80539 Munich, Germany; elena.dellera@neuropathologie.de (E.D.); kaspar.matiasek@neuropathologie.de (K.M.)

**Keywords:** leporidae, *Oryctolagus cuniculi*, spinal cord compression, myelocompression, spinal surgery, neurological, tetraparesis, intravertebral disc disease, histology

## Abstract

**Simple Summary:**

An 8-year-old rabbit presented with an acute onset of difficulty walking in all limbs. Computed tomography and magnetic resonance imaging revealed a dorsolateral cervical spinal cord compression at the level of the C6-C7 intervertebral disc. After medical treatment failure, the rabbit underwent surgical decompression of the spinal cord. The retrieved material histologically resembled degenerated and mineralized disc material. This is the first report of a cervical disc extrusion in a rabbit.

**Abstract:**

An 8-year-old rabbit presented with a 5-day history of acute difficulty in walking. Neurological examination revealed tetraparesis, proprioceptive deficits in both pelvic limbs and the right thoracic limb, decreased withdrawal reflex on the right thoracic limb and hyperreflexia in the pelvic limbs. A cervico-thoracic (C6-T2) localization was suspected. Computer tomography (CT) and magnetic resonance imaging (MRI) scans were performed, revealing a right dorsolateral extradural lesion at the C6-C7 intervertebral disc space. Additionally, meningeal and paravertebral contrast enhancement was observed on MRI, while periosteal reaction was evident at the right C6-C7 facet joint on CT. The findings were primarily consistent with spinal cord compression due to the presence of extruded disc material. Following conservative treatment failure, a right-sided C6-C7 hemilaminectomy was performed to remove the compression and sample the extradural material. Histological examination confirmed the presence of degenerated and partially mineralized disc material mixed with granulation tissue. This is the first reported case of cervical disc extrusion in a rabbit, confirmed by histological examination.

## 1. Introduction

In dogs, intervertebral disc extrusions (IVDEs) are frequently described, particularly in chondrodystrophic patients, affecting both thoracolumbar and cervical regions. In cats, IVDEs are less commonly reported [1,2,3,4,5].

Intervertebral disc extrusions and their treatment are rarely reported in rabbits. While there are some cases of thoracolumbar discopathies, cervical herniations have not been previously described to the authors’ knowledge [6,7,8,9,10,11,12].

This report details the clinical, neuroradiological, surgical, and pathological features of a histologically confirmed degenerate cervical disc extrusion in a rabbit.

## 2. Case Description

An 8-year-old male intact domestic rabbit (*Oryctolagus cuniculus*) was referred to the Centro Veterinario Gregorio VII for evaluation of a 5-day history of acute-onset gait abnormalities, without a history of previous known external trauma.

Radiography of the vertebral column of the non-anesthetized patient, conducted prior to admission by a primary care veterinarian, revealed kyphosis at the level of T7-T8, which was considered an incidental finding common in rabbits and unrelated to the observed neurological signs [8].

The general physical examination was unremarkable except for the gait abnormality. Heart and respiratory rates and body temperature were within the reference ranges. The rabbit’s body weight was 1.67 kg, and it was regularly vaccinated.

A neurological examination revealed a normal mental status; moderate ambulatory tetraparesis, with greater severity on the right side; and reduced proprioception in both pelvic limbs and in the right thoracic limb. Examination of spinal reflexes revealed hyperreflexia in the pelvic limbs; the withdrawal reflex was normal in the left thoracic limb and reduced in the right thoracic limb. The cranial nerve examination was normal. No urinary or fecal incontinence was observed [13,14]. Neurological findings were consistent with a right-sided C6-T2 spinal cord neurolocalization [13,14].

Differential diagnoses included vascular, infectious (i.e., Encephalitozoon cuniculi, Toxoplasmosis), or immune-mediated inflammatory lesions, trauma, neoplastic lesions (i.e., lymphoma), and intervertebral disc herniation [9,15]. Complete blood cell count and biochemistry were unremarkable.

The rabbit was premedicated with midazolam (1 mg/kg), dexmedetomidine (0.25 mg/kg), and methadone (0.5 mg/kg) administered intramuscularly (IM). General anesthesia was maintained through a continuous infusion rate of dexmedetomidine (10 µg/kg/h) and ketamine (50 µg/kg/h) during the whole procedure. After the procedures, dexmedetomidine was reversed with a dose of 0.25 mg/kg atipamezole IM; meanwhile, midazolam was antagonized using a dose of 0.05 mg/kg of flumazenil intravenously (IV). 

**Figure 1:**
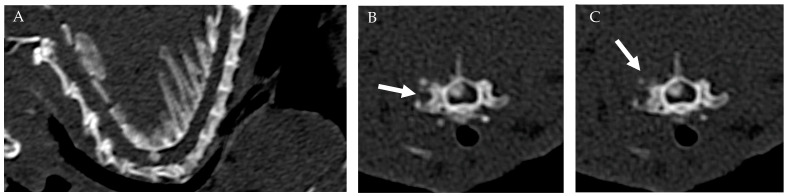
CT: Sagittal (**A**) and transverse images of the cervical vertebral column (**B**,**C**) at the level of the C6-C7 articular facet joint. An extradural well-defined, ovoid hyperdense lesion was situated dorsal–lateral (right-sided) to the spinal cord at the level of C6-C7 intervertebral space. Note the periosteal reaction surrounding the right C6-C7 articular facet joint (white arrows).

The CT scan was conducted utilizing a 16-slice multidetector CT scanner (Siemens Somatom Go Now) with the patient positioned in ventral recumbency. The acquisition was performed in the cranio-caudal direction, with a beam collimation of 0.7 mm and settings of 130 kV, 134 mAs, SAFIRE 2, and a pitch of 1 mm. Standard CT images were reconstructed using soft tissue (kernel: Br-40) and hard tissue (kernel: Br-60) filters, with a thickness of 1 mm and a reconstruction interval of 0.5 mm. The CT examination revealed an extradural hyperdense lesion situated right dorsolateral to the spinal cord at the level of C6-C7 intervertebral space (IVS), occupying approximately 50% of the spinal canal in the transverse section. The lesion appeared ovoid in shape, with well-defined margins, and no adhesion to the dorsal arch. In addition, periosteal reaction was noted surrounding the right C6-C7 articular facet joint, without evidence of fractures or osteolysis (Figure 1).

**Figure 2:**
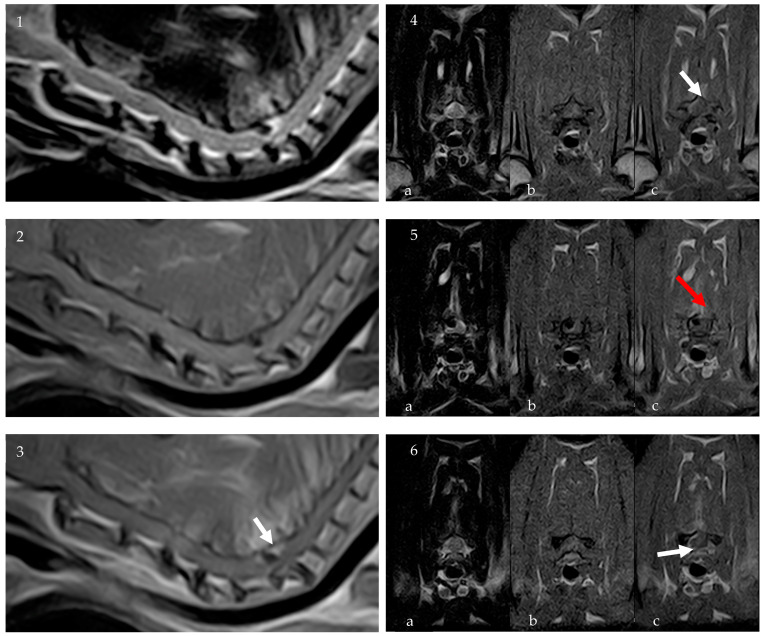
MRI: Sagittal T2W (**1**), sagittal T1W (**2**), and sagittal post-contrast T1W (**3**) images of the cervical spinal spine. Transverse images at the level of the C6 vertebral body ((**4a**) T2W, (**4b**) T1W, (**4c**) T1W post contrast), at the level of the C6-C7 IVS ((**5a**) T2W, (**5b**) T1W, (**5c**) T1W post contrast) and the C7 vertebral body ((**6a**) T2W, (**6b**) T1W, (**6c**) T1W post contrast). The MRI confirmed the presence of homogeneously hypointense material on T1W and T2W sequences, consistent with a partially mineralized extradural lesion. Post-contrast sequences revealed spinal meningeal enhancement surrounding the lesion (white arrows) and mild enhancement of the right epaxial paravertebral muscles (red arrow).

To further characterize the lesion, an 0.32 T MRI (Paramed MrJ 3300) of the cervico-thoracic spine was conducted immediately following the CT scan during the same anesthetic procedure. Pre- and post-contrast (Dotarem 0.5 mmol/mL at a dosage of 0.3 mL/kg) sagittal and transverse T1-weighted (T1W) and sagittal and transverse T2-weighted (T2W) sequences were acquired, with a slice thickness of 2.5 mm, interslice spacing of 0.28, and a matrix size of 256 × 256. The MRI confirmed the presence of homogeneously hypointense material on T1W and T2W sequences, consistent with a partially mineralized extradural lesion. The lesion, ovoid in shape with regular margins, was identified at the level of C6-C7 IVS, to the right and dorsal to the spinal cord, measuring approximately 2.5 mm × 2 mm × 4 mm. It caused severe compression of the spinal cord, estimated to affect about 50% of the cross-sectional area. There were no signs of a fracture, subluxation, or other trauma. Post-contrast sequences revealed spinal meningeal enhancement surrounding the lesion and mild enhancement of the right epaxial paravertebral muscles, particularly prominent around the right C6-C7 articular facet joint (Figure 2).

The apparent slight reduction in volume of the C6-C7 intervertebral disc (IVD) and the dorsolateral distribution of the material suggests a potential discogenic origin for the lesion, possibly associated with meningeal inflammation and muscular denervation secondary to an extrusive or traumatic event. The lack of continuity with the IVD and periosteal reaction around the articular facet joints also raised concerns about the possibility of additional differentials as an inflammatory (granulomatous or abscess-like), hemorrhagic, or neoplastic lesion (e.g., osteochondroma/sarcoma, fibrosarcoma). However, the absence of osteolytic lesions and the clinical–hematological profile make inflammatory and neoplastic differentials less likely.

The patient underwent oral therapy with meloxicam at a dosage of 1 mg/kg/day for 7 days and sulfamethoxazole and trimethoprim (30 mg/kg twice daily PO) for 8 days and cage rest. After an initial improvement, the rabbit’s neurological condition deteriorated, showing increased difficulty in walking and frequent collapses on the right side. Considering the severity as for neurological condition, it was decided that surgical decompression with sampling of the lesion would be performed.

The rabbit was premedicated with the same anesthetic protocol as for diagnostic imaging. A 26-gauge × 19 mm IV catheter was placed in the right marginal auricular vein. General anesthesia was induced with propofol (2 mg/kg) administered IV and a 3.5 mm uncuffed endotracheal tube was placed via endoscopic endotracheal intubation. Anesthesia was maintained with isoflurane in oxygen and mechanical ventilation to maintain normocapnia. Intraoperative analgesia was provided with intravenous constant-rate infusion (CRI) of fentanyl–lidocaine, consisting of 0.002 mg/kg/h fentanyl and 3 mg/kg/h lidocaine adjusted as indicated. Standard saline (0.9% NaCI) solution (10 mL/kg/h) was administered as fluid therapy. During surgery, the patient experienced a severe episode of hypotension which was responsive to norepinephrine treatment (0.5 µg/kg/min).

To perform the dorsal approach to the cervical spine, the patient was positioned in sternal recumbency. To accurately identify the C6-C7 IVS, an intraoperative fluoroscopy intensifier (Siemens Siremobil 2000; Munich, Germany) was used. After incising the skin from the spinous process of C5 to the spinous process of T1, the biventer cervicis muscles were divided along the midline raphe, and the nuchal ligament was retracted to the left side using Gelpi retractors. The epiaxial muscles were then elevated from the spinous processes and laminae using a periosteal elevator and retracted with Gelpi retractors to expose the spinous processes and laminae. To allow for accurate visualization of the area, an operating microscope was used throughout the hemilaminectomy procedure and lesion removal. A high-speed burr and a 0.5 mm Kerrison rongeur were used to perform a right-sided hemilaminectomy and expose the spinal cord. The extradural lesion was delicately extracted and any adhesions between the lesion and the dura mater and interarcuate ligament were released (Video S1). The extradural lesion was collected and submitted for histological examination. The muscles and fascial layers were sequentially apposed using absorbable material. Subcutaneous and skin layers were closed as per routine. The duration of the surgical procedure was 45 min.

After surgery, dexmedetomidine was reversed with a dose of 0.25 mg/kg atipamezole IM, and midazolam with a dose of 0.05 mg/kg of flumazenil IV. Postoperative pain control was managed with meloxicam (0.3 mg/kg) SC and methadone (0.6 mg/kg) IM. A standard saline (0.9% NaCI) solution (10 mL/kg/h) IV was administered as fluid therapy.

The patient’s recovery from anesthesia was uneventful albeit slow. After 6 h, the rabbit began to eat spontaneously and was able to walk independently, albeit with persistent tetraparesis worse on the right side. Unfortunately, during the postoperative period, the rabbit developed acute renal failure and passed away 18 h after surgery.

**Figure 3:**
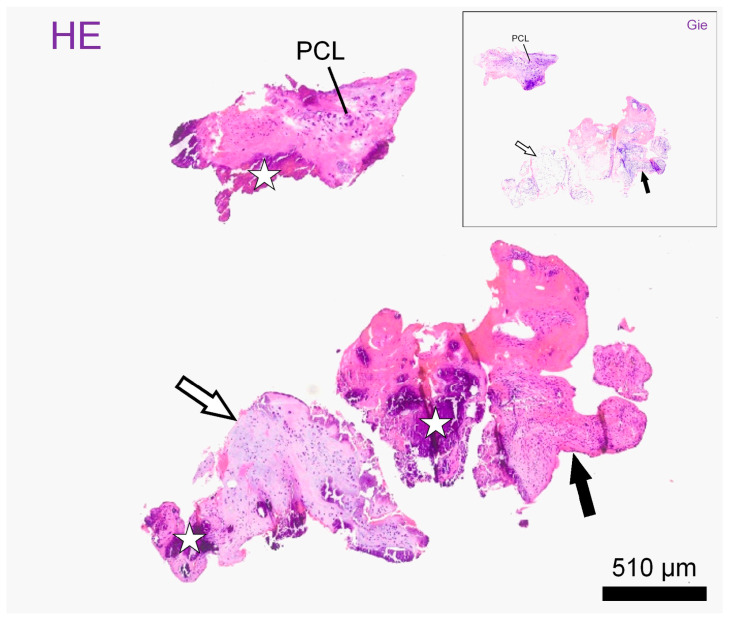
Histological slides: Representative intervertebral disc fragments on histological slides after haematoxylin–eosin stain (HE) and Giemsa stain (window: Gie). Fragments consist of hyaline (empty arrows) and fibrocartilage (black arrows) and present with atypical polycellular chondrocyte clusters (PLC) as well as with large mineralized areas, better seen on HE (stars). Scale bar: 510 µm.

Histological examination of the material on formalin-fixed paraffin embedded standard sections showed mostly fragments of mildly disorganized annulus fibrosus (outer and intermediate zones) accompanied by smaller pieces of nucleus pulposus, both exhibiting chondroid metaplasia. Both the nucleus polposus and the anulus fibrosus presented with vital chondrocyte lacunes embedded in mostly hyaline matrix. The fragments showed intravital tears accompanied by the focal proliferation of fibrovascular granulation tissue. Throughout the cartilage fragments and newly formed tissue, there were vast areas of dystrophic mineralization. The diagnosis was a space occupying the extrusion of degenerate intervertebral disc material embedded in granulation tissue (Figure 3 and Figure S1).

## 3. Discussion

Paralysis is a relatively common event in rabbits, with the most frequent cause being subluxation or fracture of the lumbar vertebrae following incorrect handling or spontaneous self-inflicted trauma [9,11,16]. Individual case reports have described articular cysts and osteomyelitis with secondary compression of the thoracolumbar spinal cord and neurological signs [17,18]. In the cervical spine, a single case of complex atlanto-axial malformation causing a compressive myelopathy was described [19]. Compared to these incidents, discopathies are rarely reported in rabbits [6,7,8,9,10,11,12]. In 1975, two of four paraplegic cases in a case series were reported to have IVDEs diagnosed post mortem [10]. In a review of rabbits’ neurological disorders, IVDEs are described as rare events associated with a poor prognosis [9]. One study described a successfully surgically treated thoracolumbar IVDE in a paraplegic rabbit [6]. Meanwhile, a recent study described the successful conservative treatment of recurrent thoracolumbar IVDEs in a rabbit with paraparesis [12]. To our knowledge, this is the first described case of cervical IVDE in a rabbit. 

Intervertebral disc extrusions are a frequent cause of neurological disorders in dogs, especially in chondrodystrophic breeds, and less commonly in cats [1,2,5,20]. The limited description of discopathies in rabbits could result from the infrequent utilization of advanced imaging diagnostics, prompted by both economic and anesthesia-related risks and concerns [21]. The recent increase in descriptions of IVDEs in rabbits could be attributed to the increasing use of MRI, coupled with advancements in machine technology and imaging protocols [6,12]. These advancements have made it easier to obtain high-quality diagnostic images even in smaller patients. Prompt diagnosis of intervertebral disc extrusions could enable targeted and timely implementation of conservative or surgical treatments, potentially preventing neurological decline and improving patient outcomes.

In our rabbit, the histological examination showed dystrophic disc degeneration with chondroid metaplasia of the nucleus pulposus. The granulation tissue highlighted in this histological examination is a frequent finding in IVDEs, especially in association with calcified material. It is caused by an inflammatory reaction triggered by the presence of the nucleus pulposus in the extradural space [22,23]. In our rabbit, the signal characteristics of the extruded material on MRI and CT are suggestive of mineralized material, which would confirm dystrophic degeneration. Similar to our rabbit, the herniated material in the case reported by Della Camera was hypointense in both the T1W and T2W sequences, indicating signal characteristics consistent with degenerated disc material [12]. Intervertebral disc extrusion in dogs is often associated with IVD degeneration in chondrodystrophic patients. In rabbits, the true frequency of these events is unknown, as well as whether there is a correlation with chondrodystrophy. In our patient, the extrusion of this material could be due to a traumatic event not witnessed by the owners. Such a possibility would justify both the dorsal migration of the material and the hyperostotic reaction affecting the articular facet joint as well as the acquisition of contrast medium by the paravertebral muscles. The CT images did not reveal a facet fracture, but given the small size of the patient, it is not possible to completely rule out a microfracture.

In our case, we initially opted for a spinal CT scan to minimize anesthesia time. The CT showed a lesion in the vertebral canal with a periosteal reaction at the C6-C7 articular facet joint. A CT myelogram would not have provided better characterization of the lesion, as it would not have allowed an assessment of the surrounding soft tissues or spinal cord; therefore, we opted for MRI [24]. Despite the small size of the patient, it was possible to perform a complete study of the cervico-thoracic spine with good spatial resolution.

The MRI confirmed the presence of an extradural lesion, with signal characteristics primarily consistent with calcified material. Additionally, it highlighted meningeal and paravertebral contrast enhancement, alterations already described in dogs with IVDEs [25,26,27]. 

Initially, the rabbit underwent conservative treatment, showing some improvement with meloxicam and sulfamethoxazole and trimethoprim, but unfortunately, the rabbit deteriorated, displaying progressive difficulty in walking and frequent falls on the right side. The failure of conservative therapy is likely attributable to the extent of spinal cord compression. Considering the worsening neurological signs, surgical decompression was further pursued.

The extruded material was successfully removed during surgery, despite adhesions with the dura mater and the interarcuate ligament. Within a few hours post-surgery, the patient was able to stand and take a few steps, exhibiting a neurological condition comparable to pre-surgery status. This finding suggests that rabbits can safely undergo cervical spinal cord decompressive surgery. The patient tolerated CT and MRI anesthesia without complications. Despite the short surgical duration, the patient unfortunately experienced a severe episode of intraoperative hypotension. Since effective measures for managing intraoperative hypotension in rabbits are not described in the literature, we employed the most effective procedures described in other domestic animals. However, this event likely resulted in renal failure and subsequent death during the post-operative period. Consequently, close monitoring of hemodynamics and enhanced post-operative care are crucial to reduce the risk of renal complications in rabbit surgery. Due to these complications, it was not possible to evaluate the outcome of spinal decompression in our rabbit.

## 4. Conclusions

While a larger sample size is required to accurately determine the prevalence of rabbit IVDEs, particularly in the cervical region, and their potential association with traumatic events or chondrodystrophy, our results suggest that IVDEs should be considered in the differential diagnosis of rabbits presenting with acute tetraparesis [28,29]. Consequently, despite the inherent risks associated with the anesthetic procedure in rabbits, we advocate for comprehensive diagnostic evaluations given that IVDEs represent a benign pathology possibly responsive to surgical intervention.

## Data Availability

This article includes all relevant study data.

## Supplementary Materials

The following supporting information can be downloaded at: Video S1: Hemilaminectomy (https://doi.org/10.5281/zenodo.11408624, accessed on 18 July 2024); Figure S1: Histological slides (https://doi.org/10.5281/zenodo.12664402, accessed on 18 July 2024).
